# Protein kinase C and rho activated coiled coil protein kinase 2 (ROCK2) modulate Alzheimer's APP metabolism and phosphorylation of the Vps10-domain protein, SorL1

**DOI:** 10.1186/1750-1326-5-62

**Published:** 2010-12-30

**Authors:** Rachel F Lane, Joshua W Gatson, Scott A Small, Michelle E Ehrlich, Sam Gandy

**Affiliations:** 1 Department of Neurology, Mount Sinai School of Medicine, New York NY 10029, USA; 2 Department of Surgery, University of Texas Southwestern, Dallas TX 75390, USA; 3 Department of Neurology, Taub Center for Research on the Aging Brain, Columbia University College of Physicians and Surgeons, New York NY 10032, USA; 4 Department of Pediatrics, Mount Sinai School of Medicine, New York NY 10029, USA; 5 Department of Genetics and Genomic Sciences, Mount Sinai School of Medicine, New York NY 10029, USA; 6 Department of Psychiatry, Alzheimer's Disease Research Center, New York NY 10029, USA; 7 James J Peters VA Medical Center, Bronx NY 10468, USA

## Abstract

**Background:**

Generation of the amyloid β (Aβ) peptide of Alzheimer's disease (AD) is differentially regulated through the intracellular trafficking of the amyloid β precursor protein (APP) within the secretory and endocytic pathways. Protein kinase C (PKC) and rho-activated coiled-coil kinases (ROCKs) are two "third messenger" signaling molecules that control the relative utilization of these two pathways. Several members of the Vps family of receptors (Vps35, SorL1, SorCS1) play important roles in post-*trans*-Golgi network (*TGN*) sorting and generation of APP derivatives, including Aβ at the TGN, endosome and the plasma membrane. We now report that Vps10-domain proteins are candidate substrates for PKC and/or ROCK2 and act as phospho-state-sensitive physiological effectors for post-*TGN *sorting of APP and its derivatives.

**Results:**

Analysis of the SorL1 cytoplasmic tail revealed multiple consensus sites for phosphorylation by protein kinases. SorL1 was subsequently identified as a phosphoprotein, based on sensitivity of its electrophoretic migration pattern to calf intestine alkaline phosphatase and on its reaction with anti-phospho-serine antibodies. Activation of PKC resulted in increased shedding of the ectodomains of both APP and SorL1, and this was paralleled by an apparent increase in the level of the phosphorylated form of SorL1. ROCK2, the neuronal isoform of another protein kinase, was found to form complexes with SorL1, and both ROCK2 inhibition and ROCK2 knockdown enhanced generation of both soluble APP and Aβ.

**Conclusion:**

These results highlight the potential importance of SorL1 in elucidating phospho-state sensitive mechanisms in the regulation of metabolism of APP and Aβ by PKC and ROCK2.

## Introduction

Aberrant processing of the Alzheimer's amyloid precursor protein (APP) is believed to underlie some forms of Alzheimer's disease (AD), leading to increased generation and/or decreased clearance of amyloid beta 42 (Aβ42). APP is differentially processed within discrete intracellular compartments. Metabolism of APP by either the endocytic pathway or the constitutive secretory pathway is regulated on a moment to moment basis by the integration of intercellular and intracellular signals, including membrane depolarization and first messenger activation of their cognate receptors [for reviews, see 1-3]. Second messengers such as calcium and cyclic AMP act via third messengers that are enzymes that control protein phosphorylation (i.e., protein kinases and protein phosphatases). Third messengers enzymes implicated in regulating APP metabolism include protein kinase C (PKC; [4-9]), protein phosphatases 1 and 2A (PP1, PP2A; [4-7]), extracellular signal regulated protein kinase (ERK; [10]), casein kinases (CK; [11]), Janus kinase (JNK; [12,13]), and rho-associated coiled-coil protein kinases (ROCK; [14]).

We have a longstanding interest in identifying the important phospho-state-sensitive physiological effectors that are targets for these third messengers [4,8,15]. The obvious candidates for identities of phospho-state sensitive molecules relevant to APP metabolism include: (i) APP itself [4,15]; (ii) the various APP sorting and trafficking proteins ([8]; this manuscript); and (iii) the secretases [16,17]. APP phosphorylation at serine 655 was discovered in 1988 [4], and its physiological role includes regulation of the interaction of APP with the retromer trafficking complex [15] and activation of PKC is associated with increased retromer-mediated transport of APP to the *TGN *and decreased Aβ generation [15]. In addition to phosphorylation of APP at serine 655, additional phospho-acceptor sites have been discovered. Suzuki and colleagues have documented an important role of the phosphorylation state of APP threonine 668 in APP maturation and sorting [reviewed in [3]] while phosphorylation of the cytoplasmic tail at tyrosine 682 and/or 687 has been reported to regulate release of the APP intracellular domain (AICD; [18,19].

APP retrograde trafficking to the *TGN *and regulation of Aβ generation is dependent on SorL1 interaction with the core component of the retromer complex, Vps35 [20,21]. In addditon, we have recently implicated a second member of the Vps10 family, SorCS1 in retromer-mediated regulation of Aβ generation [22]. In the current study, we have begun investigating the possibilities that some of these Vps10-domain proteins are also important phospho-state-sensitive modulators of post-*TGN *APP metabolism. Herein, we report: (i) that SorL1 is a phosphoprotein; (ii) that both PKC and ROCK2 interact with SorL1; and (iii) that modulation of ROCK2 activity regulates generation of Aβ.

## Results and Discussion

### The subcellular trafficking itinerary for APP

In order to understand how PKC, ROCK, and SorL1 might play important roles in APP metabolism, one must understand the details of APP sorting and processing in post *TGN *compartments. Following exit of mature APP from the *TGN*, some APP molecules are conveyed by the secretory pathway to the plasma membrane where APP can encounter and be cleaved within the Aβ domain by one of the α-secretases; this is the nonamyloidogenic (non-Aβ-forming) pathway) (for review see, [2]). α-secretase cleavage of APP results in the formation of a cell retained carboxyl terminal fragment (α-CTF) and in the shedding of the APP ectodomain, which is also known as soluble APPα (sAPPα). APP molecules that do not enter the secretory pathway are targeted to a *TGN*-localized population of clathrin-coated vesicles (CCVs). From here, APP is conveyed into the endocytic pathway where APP can encounter and be cleaved by the β-secretase, BACE (for β-APP site cleaving enzyme). BACE cleavage of APP occurs primarily in endosomes, and this step defines APP entry into the potentially amyloidogenic (Aβ-forming) pathway, resulting in formation of a soluble sAPPβ fragment and a membrane bound β CTF, amino terminus of which is identical to that of Aβ [for review, see [2]]. APP not cleaved at the plasma membrane by the α-secretases can be reinternalized into the endocytic pathway thereby also entering the amyloidogenic pathway.

Within the endosomal system, the α- and β-CTFs serve as the proximate substrates for γ-secretase. γ-secretase cleavage of the α-CTF gives rise to secreted nonamyloidogenic p3 and an intracytoplasmic fragment, AICD (APP intracellular domain), whereas γ-secretase cleavage of the β-CTF gives rise to Aβ and also to AICD. The γ-secretase cleavage is especially important since this is the step where the major speciation occurs, giving rise to the heterogeneous carboxyl termini that define Aβ40 and Aβ42. Oligomerization and accumulation of Aβ42 are believed to be the key inciting events in causation of AD. Ultimately reducing the time of residence of APP and/or the β-CTF within the endocytic pathway decreases Aβ generation. Retromer-mediated trafficking of APP and/or its CTFs away from the endosome to the TGN plays a key role in modulating residence time for APP and/or its CTFs in the endocytic system [for review, see [2]]. As shown by Small *et al *[20], the retromer is especially important in sporadic AD because hippocampal subregion-specific progression of AD pathology is linked to a deficiency in the core component of the retromer, Vps35 [20].

Post-*TGN *sorting and metabolism of APP is controlled by at least two Vps10 domain proteins, SorL1 [2,23-27] and SorCS1 [22]. At least one action of SorL1 involves its function as a retromer adaptor or receptor, linking APP to Vps35 [2,21]. When the SorL1-APP interaction is abolished by mutation of the FANSHY domain in the cytoplasmic tail of SorL1 that specifies its binding to Vps35, that FANSHY domain mutant is unable to regulate APP metabolism [21]. In addition to its role in retromer-mediated retrieval of APP, another model suggests that SorL1 plays a gating or retention function for APP at the *TGN *[27]. The molecular mechanism for this interaction at the *TGN *has been suggested to involve SorL1 and APP interacting together with the adaptors GGA and PACS-1 to control exit of APP into the amyloidogenic and non-amyloidogenic pathways [27]. The two models for SorL1 action (the retromer model and the *TGN *retention model) are not mutually exclusive, and their relative importance remains unknown.

### Regulation of APP sorting and metabolism by protein phosphorylation

The first and second messengers linked to APP processing lead to activation of third messenger protein kinases and protein phosphatases. PKC and ROCK have emerged as especially interesting since they have different effects on APP metabolism, perhaps due to differential effects on sorting of APP and/or its derivatives. PKC activates the α-secretase pathway [4-8], while ROCK inhibits the α-secretase pathway [14]. These protein kinases and protein phosphatases converge on physiological effector substrate phosphoproteins, and the phosphorylation status of those effectors typically specifies their states of activation. Identification of the phospho-state sensitive effector molecules that modulate APP metabolism has been challenging. Vps10-domain proteins are outstanding candidates for such effectors. Since SorL1 has been proposed [2] and recently demonstrated [21] to act as an adaptor that links APP to the retromer complex, we began our study of phospho-state regulated APP metabolism by Vps10 domain proteins with the current study of SorL1.

### SorL1 is a phosphoprotein

**Figure 1:**
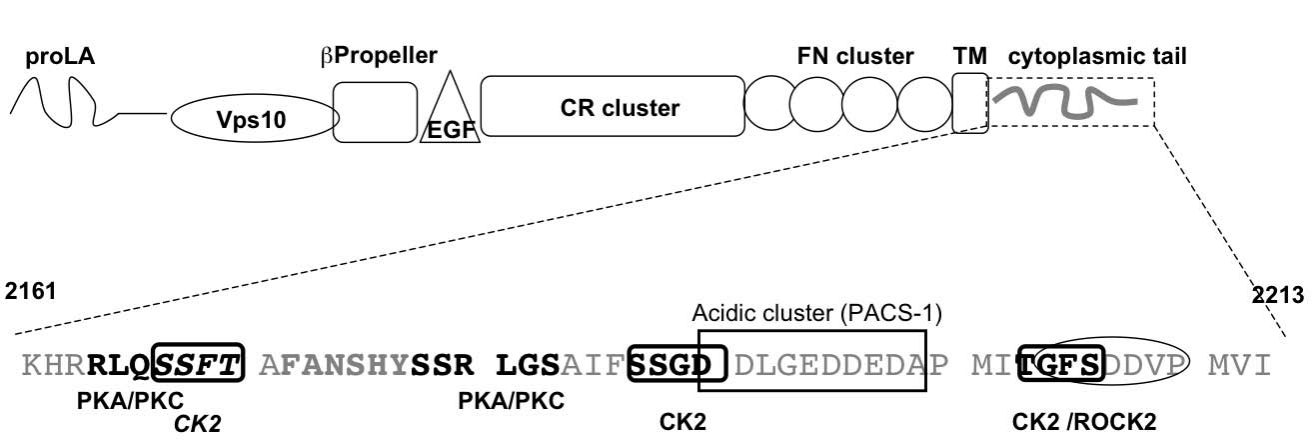
**The SorL1 cytoplasmic tail contains multiple putative kinase recognition motifs**. Analysis of the SorL1 cytoplasmic domain using phospho-motif software that reports the presence of literature-derived consensus recognition motifs for protein kinases [28] revealed multiple putative phosphorylation sites. Recognized motifs include three for PKC/PKA [R/K XX S/T] [S/T X R/K] at 2167, 2183 and 2178 (**bold black**) and three for CK2 [S/T XX E/D] at 2167, 2187, 2206 (**circled bold black**) and one for ROCK2 (circled).

Phospho-site consensus motif software that identifies the presence of known consensus kinase recognition motifs [28] revealed multiple putative phosphorylation sites in the SorL1 cytoplasmic tail (Figure 1). As mentioned above, in addition to these phospho-acceptor consensus motifs, a motif in the SorL1 cytoplasmic tail has been recently identified that is essential for its interaction with Vps35 [21]. This motif, 2172 Phe-Ala-Asn-Ser-His-Tyr 2173 (FANSHY), is flanked by consensus sites for PKC phosphorylation at serines 2167 and 2178. The FANSHY domain is of particular relevance since the mutations of this motif that abolish SorL1-Vps35 interaction also abolish the ability of SorL1 to control APP processing [21]. These observations raised the possibility that the phosphorylation status surrounding the FANSHY domain might play a key role in the modulation of APP metabolism by SorL1. In order to begin to test this hypothesis, we first sought to determine whether SorL1 is itself a phosphoprotein.

**Figure 2:**
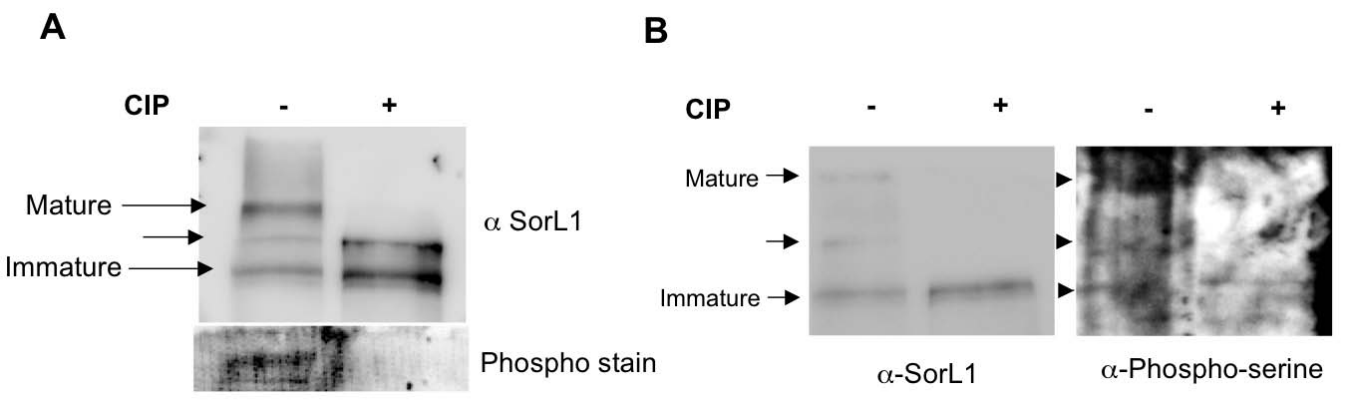
**SorL1 is a phosphoprotein**. Wt MEFs were grown to confluency and equal concentrations of protein lysate were incubated with (+) or without (-) calf intestine alkaline phosphatase (CIP). Equal volumes were subsequently analyzed in parallel by immunoblotting with either; α-SorL1 antibody (BD biosciences), phosopho-serine or ProQ diamond phospho-stain (Invitrogen). **A**. Separation of samples by 6% SDS PAGE resulted in identification of multiple SorL1 immunopositive bands (-). Following treatment with CIP (+) (first panel) the largest molecular weight SorL1 immunopositive band was seen to decrease in molecular weight and as a consequence migrate with the two faster migrating bands. Detection with ProQ diamond phospho stain that selectively stains phosphoproteins in polyacrylamide gels detected a signal in only the untreated sample (-) as detected by UV transillumination (second panel). **B**. Immunoblotting of independent CIP treated lysates revealed phospho-serine immunopositive bands (right panel) with the same migratory pattern as SorL1 positive bands. To confirm that the phospho-serine antibody was cross-reacting with SorL1, the phospho-serine immunoblot was stripped and reprobed with α-SorL1 antibody

To determine whether SorL1 is a phosphoprotein, we investigated the electrophoretic migration pattern of SorL1 on low percentage SDS PAGE before and after dephosphorylation. SorL1 immunopositive bands following a mock reaction without calf intestine alkaline phosphatase (CIP) revealed three SorL1 immunopositive species migrating at approximately 250-270 kDa that were independently detected with two different anti-SorL1 antibodies. Upon dephosphorylation with CIP, the electrophoretic migration pattern shifted dramatically, and the highest molecular weight species of SorL1 shifted and co-migrated with the lower molecular weight, immature species (Figure 2A). This behavior was consistent with the possibility that SorL1 is a phosphoprotein, and that the slower migrating SorL1 species was modified by the addition of one or more phosphate moieties.

In order to seek independent confirmatory evidence that SorL1 is a phosphoprotein, we employed the ProQ Diamond phospho-stain (Invitrogen) that specifically detects phosphorylated proteins in polyacrylamide gels. ProQ detected multiple bands of approximately 250 kDa that co-migrated with the high molecular weight candidate phospho-SorL1, and these were abolished following treatment with CIP (Figure 2A).

We also sought to determine whether the high molecular weight candidate phospho-SorL1 could be detected with a specific phospho-serine antibody. Immunoblotting of untreated and CIP-treated lysates revealed immunopositive bands with the same migratory pattern as SorL1. As before, the higher molecular weight SorL1 species were not detected with this antibody following dephosphorylation with CIP (Figure 2B). Taken together, the altered migration of SorL1 following dephosphorylation and the staining with both a phospho-stain and with anti-phospho-serine antibodies support the conclusion that SorL1 is a phosphoprotein.

### PKC activation stimulates SorL1 phosphorylation

**Figure 3:**
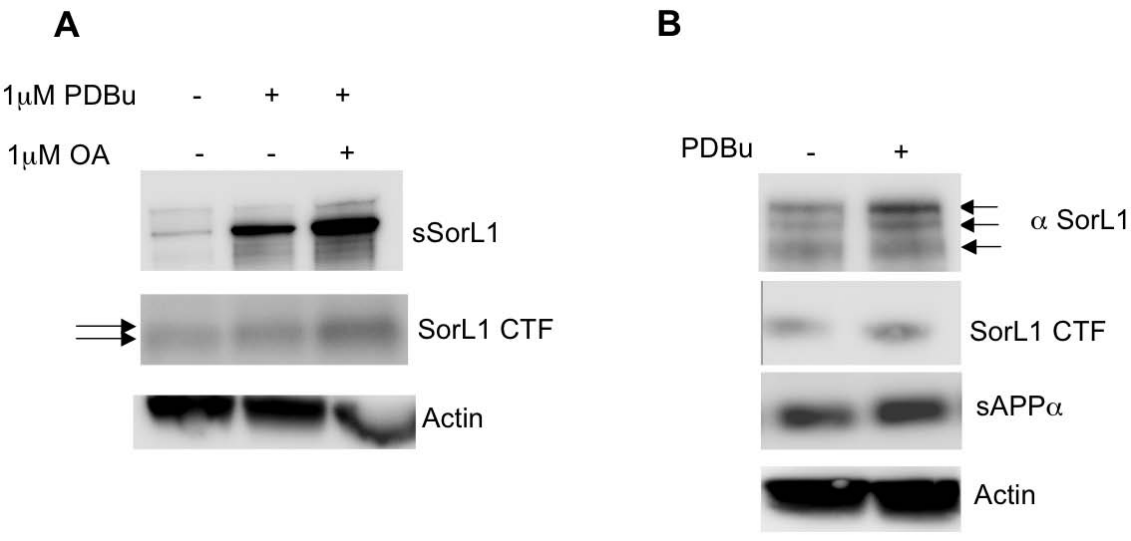
**Activation of PKC stimulates SorL1 ectodomain shedding and SorL1 phosphorylation**. Wt MEFs were grown to confluency and incubated for 10 min in the presence of vehicle (-), 1 uM PDBu and/or 1 uM Okadaic acid (OA) (+). Equal volumes of media and protein lysate were subjected to SDS PAGE, transferred to PVDF and probed with α-SorL1 (N-terminal BD Biosciences) to detect holo SorL1 and the soluble SorL1 (sSorL1) shed ectodomain, or α-SorL1 C-terminus (J. Lah, Emory) to detect SorL1 CTFs and α-actin as a loading control. **A**. Treatment with 1 μM PDBu (PKC activator) alone and in combination with 1 uM Okadaic acid, increased ectodomain shedding of SorL1 (sSorL1) and sAPPα into the media and increased SorL1 cell associated CTF generation. The migration of the SorL1 CTF appeared slightly retarded upon stimulation of PKC and inhibition of PP1 and PP2A. **B**. Treatment of lysates with 1 μM PDBu increased levels of the largest molecular weight immunopositive SorL1 species.

PKC activates α-secretase-mediated ectodomain shedding of APP, and a number of γ-secretase substrates, including the Vps10 family members sortilin, SorL1, SorCS1, 2 and 3 [8,23]. Therefore, we sought to determine whether SorL1 phosphorylation was enhanced following stimulation of PKC. As previously reported, phorbol-12,14-dibutyrate (PDBu, Sigma), a PKC activator, stimulates ectodomain shedding of APP and other γ-secretase substrates including SorL1 [8,23] (Figure 3A). Treatment of mouse embryonic fibroblasts (MEFs) with PDBu increased shedding of the ectodomains of SorL1 and APP, and that effect was greater following treatment with the combination of PDBu and okadaic acid (OA), an inhibitor of protein phosphatases 1 and 2A (Figure 3A, B). Interestingly, upon treatment with PDBu, the highest molecular weight SorL1 species (upper two bands) showed increased signal intensity (Figure 3B) and migration of the SorL1 CTF was slightly retarded compared to untreated control (Figure 3A), consistent with the shifts expected for proteins following addition of phosphate moieties.

Taken together with our previous observations, these data suggest that PKC activation results in increased phosphorylation and ectodomain shedding of SorL1. We cannot yet determine how SorL1 shedding influences its ability to regulate Aβ. Further study is required to determine whether PKC does indeed directly phosphorylate SorL1, and, if so, whether this addition of phosphate to SorL1 is essential for regulation of soluble SorL1 and APP shedding and/or Aβ generation. The role of the proximity of the FANSHY domain to the potential phosphorylation sites also remains to be explored.

### SorL1 interacts with ROCK2

**Figure 4:**
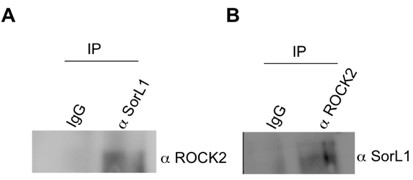
**SorL1 interacts with ROCK2**. **A**. Endogenous SorL1 was immunoprecipitated from Wt MEFs using α SorL1 antibody (BD biosciences) or control IgG. Immunooprecipitates were subjected to SDS-PAGE, transferred to PVDF membranes and probed α-ROCK2. ROCK2 was detected in the α-SorL1 precipitate. **B**. Immunoprecipitation of ROCK2 using α-ROCK2 antibody (BD biosciences) resulted in co-immunoprecipitation of SorL1.

We have previously reported that ROCK1 inhibits basal and statin-activated α-secretase cleavage and shedding of sAPPα [14]. While ROCK1 is highly expressed in lung, heart, stomach, liver, kidney, placenta, and testis, it is the ROCK2 isoform that is more abundant in the heart and brain [29], suggesting that the actions of ROCK in the brain may be mediated primarily by the ROCK2 isoform. We investigated the possibility that ROCK2 interacts with SorL1. Immunoprecipitation of SorL1 resulted in co-immunoprecipitation of ROCK2 (Figure 4A). To confirm this interaction, we performed the reverse immunoprecipitation and, indeed, SorL1 was recovered from the anti-ROCK2 immunoprecipitate but not from the control IgG immunoprecipitate (Figure 4B). Though we did not assess whether ROCK2 could regulate phosphorylation of SorL1, Herskowitz *et al *(2010) [30] recently reported that ROCK2 modulates SorL1 phosphorylation, consistent with our observation that the two molecules can exist as a complex. As with PKC phosphorylation of the SorL1 cytoplasmic tail, the possible phospho-state-sensitive modulation of FANSHY-mediated SorL1-Vps35 interaction remains to be elucidated. Given their differential effects on APP metabolism [[14], this paper], it is tempting to speculate that differential phosphorylation of SorL1 by PKC and/or ROCK1 may be involved.

### Inhibition of ROCK2 increases Aβ generation

**Figure 5:**
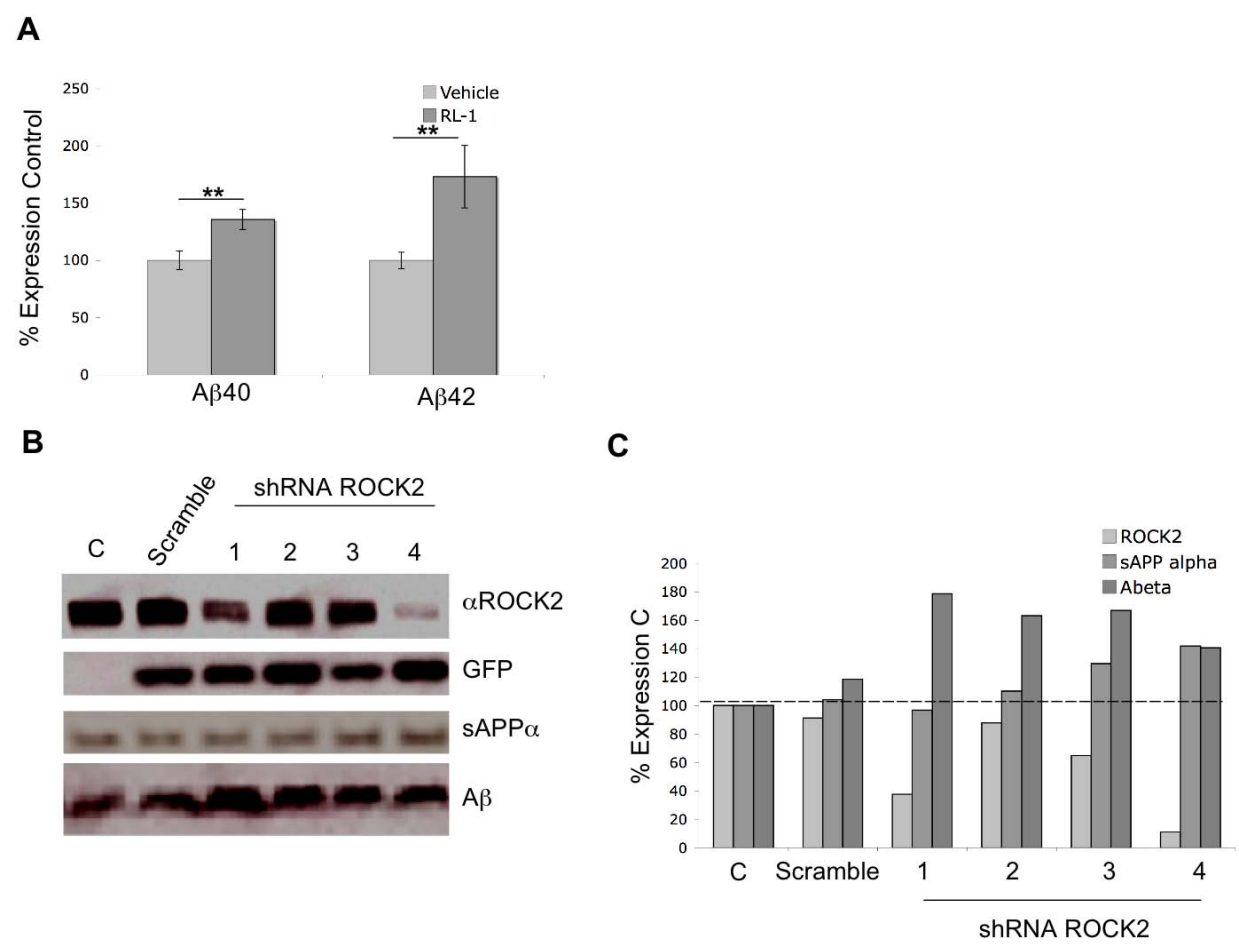
**Inhibition of ROCK2 increases Aβ40 and Aβ42 generation**. **A**. N2A APPSweΔ9PS1 cells were grown to confluency and treated with vehicle or 100 nM RL-1 for 60 mins. After 60 mins equal volumes of media were collected and Aβ40 and Aβ42 levels were determined by sandwich ELISA (Wako). Aβ40 (*p *= 0.007) and Aβ42 (p = 0.03) levels were increased following treatment with 100 nM of the specific ROCK2 inhibitor, RL-1. Data was collected in duplicate or triplicate from 3 independent iterations (*p > 0.05 **p < 0.01). **B**. N2A APP SweΔ9PS1 cells were transduced with non-specific scrambled shRNA or 4 different shRNA ROCK2. 72 hrs post transduction media and cell lysates were collected and snap frozen. Equal volume and concentrations of media and lysate respectively were analyzed by SDS PAGE and transferred to PVDF. Lysates were analyzed for ROCK2 and GFP as a control for transduction efficiency and media analyzed for sAPPα and Aβ. Protein levels are expressed as percentage of non-transduced C (C). Knockdown of ROCK2 resulted in increased generation of Aβ and sAPPα with all shRNA ROCK2 viruses analyzed but not after transduction with the scrambled non-specific control shRNA.

Having identified ROCK2 as a SorL1 binding protein, we next sought to determine the effect of ROCK2 activity on Aβ generation. Treatment of mouse N2a APPSweΔ9PS1 neuroblastoma cells for 100 mins with the selective ROCK2 inhibitor RL1 (100 nM) resulted in increased generation of both Aβ40 (*p *= 0.007) and Aβ42 (*p *= 0.03) (Figure 5A). To confirm this observation and rule out off-target affects of RL1, we next assessed the effect of specific shRNA lentiviral mediated knockdown of ROCK2. Transduction with shRNA ROCK2 (but not with the scrambled shRNA control) resulted in increased generation of both Aβ and sAPPα in 4 different shRNA ROCK2 reactions (Figure 5B, C).

Like most protein kinases, ROCK autophosphorylates itself, and the phospho-form is the more active form. The absence of perfect dose-dependence between ROCK2 knockdown and Aβ generation in Figure 5 may be due to differences in ROCK2 specific activity related to differences in phosphorylation status. Phospho-ROCK2-specific antibodies are being developed to clarify this point.

While the inhibitor and shRNA sequence used in this study are specific for ROCK2 when known substrates are studied, the two isoforms show significant homology, and we therefore cannot completely rule out non-specific effects and/or effects mediated by ROCK1.

## Conclusions

The involvement of the Rho/ROCK pathway in AD has been reported by several research groups [14,31,32]. However, the mechanisms by which ROCK regulates APP processing and Aβ generation are not well understood. While we previously demonstrated that expression of a dominant negative (DN) ROCK1 increased sAPPα, Aβ generation was not addressed in that system [14]. In the current study, we observed that shRNA mediated knockdown of ROCK2 resulted in increased release of both sAPPα and Aβ. While the increase in sAPPα is consistent with our earlier ROCK1 DN data [14] the increase in Aβ is unexpected. One explanation may lie in the mechanism and subcellular localization by which SorL1 regulates Aβ generation. This is the first example of a protein kinase that regulates both α- and β-secretase pathways in parallel. These data can be construed to fit either the TGN model of SorL1 action or the retromer model. SorL1-phospho-state-dependent dissociation of APP and/or CTFs bound in complexes localized to either the *TGN *or to the retromer would be expected to increase substrate available for generation of sAPPα and Aβ. However, the net effect on APP metabolism would also have to take into account any actions related to the phosphorylation status of APP serine-655 [15]. Additional data will be required in order to develop a model that takes into account all relevant molecules and their phosphorylation status.

Further investigation is required to establish the roles of ROCK2, PKC and various Vps10 domain proteins in the phospho-state sensitive generation of Aβ generation. Work by Small *et al *(2005)[20], Muhammad *et al*., (2009)[33], and Andersen *et al*., (2010)[21] have demonstrated the importance of the retromer, and it will be important to elucidate the possible role of direct phosphorylation of Vps35 and Vps10-domain receptors. Ultimately the regulation of APP metabolism by protein phosphorylation provides an indirect pathway for therapeutic modulation of Aβ generation. The recent evidence that Aβ plays a physiological role in normal memory formation and the recent failure of a γ-secretase inhibitor in phase 3 clinical trials has pointed up the necessity for fine regulation of Aβ metabolism in order to balance the requirement for Aβ levels that support memory function against the therapeutic goal of avoiding Aβ accumulation. Targeting a protein phosphorylation regulatory pathway for APP metabolism may enable finer adjustment than that achievable by direct inhibition of β- or γ-secretase.

Balancing between α and β secretase pathways physiology and pathophysiology will be especially important given the likelihood that Aβ-lowering drugs may well be administered prophylactically to people with intact cognition and then maintained for several decades.

## Materials and methods

### Antibodies

α-SorL1 (N-terminal, BD Biosciences), α-ROCK2 (BD-Biosciences), α-actin (Sigma), α-phospho-serine antibody (Abcam), α-GFP (Sigma), 6E10 (Covance, sAPPα and Aβ), anti-mouse, anti-rabbit HRP conjugates (Vector Labs) were purchased. α-SorL1 (C-terminal, gift from J. Lah, Emory University) to detect SorL1 C-terminal fragments (CTFs), Ab369 (C-terminal APP antibody) to detect human holoAPP and C-terminal fragments [5] were kindly donated. Pro-Q Diamond phosphoprotein Gel stain (Invitrogen) was used to detect putative phosphorylated proteins as per the manufacturers instructions.

### Cell culture studies

Human embryonic kidney 293 cells and wild type (Wt) mouse embryonic fibroblasts (MEF) cells were cultured at 37°C/5%CO_2 _in growth medium (DMEM, 10%FBS, 1% penicillin/streptomycin, 1%L-glutamine Gibco). Mouse N2a neuroblastoma cells stably co-transfected with the FAD mutant forms of APP and PS1 (N2a APPSweΔ9PS1) (gift from G. Thinakaran, University of Chicago, Chicago, IL.), were maintained in DMEM supplemented with 10% FBS, penicillin (100 U/ml), streptomycin (100 mg/ml), geneticin (200 mg/ml), and puromycin (5 mg/ml).

Wildtype MEFs were used for SorL1 phosphorylation and processing studies, N2a APPSwe ΔE9 PS1 were used for investigation for pharmacological inhibition of ROCK2 and shRNA knockdown studies and HEK 293t cells were used to generate scramble shRNA and ROCK2 shRNA lentivirus.

### Pharmacological studies

Wildtype MEFs were treated in serum free DMEM with either PDBu (PKC activator, Sigma), okadaic acid (OA) (protein phosphatase 1 (PP1) and 2A (PP2A), Sigma) or 4,5,6,7-Tetrabromobenzotriazole (TBB) (CK2 inhibitor Tocris Biosciences). At specified time points media was collected and snap frozen. Cells were harvested in RIPA buffer (50 mM Tris HCl pH 7.5, 100 mM NaCl, 1 mM EDTA, 1 mM DTT, 1% NP40, 0.2 mM PMSF, 0.2 mM Na_3_VO_4_, 50 mM NaF, 10 mM Na_4_P_2_O_7 _plus Roche complete EDTA-free protease inhibitor tablet) using 5 cycles of 20 s vortex/5 min ice incubation, with the exception of alkaline phosphatase (CIP) studies where protein phosphatases were excluded from the RIPA buffer. Cell debris was removed by centrifugation at 4°C at 10,000× *g *for 15 min.

Protein concentrations from cell lysates and media were determined using the Bio-Rad Protein Determination Kit. Absorbance was read at 595 nm using a Bio-Rad Microplate Reader (680XR) and analyzed using Microplate Manager v5.2.1. Samples were subsequently prepared in 5× Laemmli buffer and boiled at 95°C for 5 minutes. For analyses of different SorL1 immunoreactive species, lysates and media were separated by tris-glycine 6% and 10% SDS PAGE gels respectively, transferred to PVDF and immunoblotted using α-SorL1 (BD biosciences) which recognizes an epitope in the SorL1 shed ectodomain to detect 1) holo-SorL1 in lysates and 2) soluble SorL1 (sSorL1) in media samples and α-SorL1 (J. Lah, Emory) to detect the SorL1 CTF.

To detect putative phosphorylated immunopositive SorL1 species, 60 μg protein lysate was treated with vehicle or CIP (NEB) according to manufacturers instructions and subsequently prepared in 1X Laemmli buffer and analyzed as above.

N2a APPSwe PS1 were grown to 70% confluency, treated with 100 nM of the selective ROCK2 inhibitor RL-1 or vehicle control (DMSO) for 60 minutes in serum free DMEM. At 60 mins post treatment, equal volume of media were collected and snap frozen and Aβ40 and Aβ42 levels were determined by the human Aβ (40 and 42) ELISA kit (WAKO), according to the manufacturer's instructions. Absorbance was read at 450 nm using a BioRad microplate reader. Results were normalized to control (pg/mol) and expressed as percentage expression of control.

### Immunoprecipitations

Wildtype MEFs were grown to confluency and lysates were prepared as described in the previous section. A 300 μg aliquot of cell lysate was used for immunoprecipitation using A/G plus agarose beads (Santa Cruz) with either (a) 2 μg of the appropriate primary antibody (α-ROCK2, α-SorL1) or (b) IgG control antibody according to the manufacturers instructions. Equal volumes of IgG and α-SorL1/αROCK2 immunoprecipitation were separated by SDS PAGE, transferred to PVDF and immunoblotted with α-SorL1 (BD Biosciences) and α-ROCK2 antibodies as indicated.

### Lentivirus production and transduction of N2aAPPSwe Δ9PS1 cells

Lentivirus was produced in the HEK 293 cells as previously described [34], with a few modifications. In brief, the HEK cells were plated onto twelve, 15 cm petri-plates in DMEM supplemented with 10% FBS, penicillin, and streptomycin. The cells were grown until 60% confluent and co-transfected with three plasmids. Per petri-plate, approximately 57 μg of plasmid DNA was used for the transfection: 19 μg of the CMV-driven HIV gag-pol, tat and rev plasmid [35], 13 μg of the CMV-driven vesicular stomatitis virus-G envelope (VSV-G) plasmid (plasmid kindly provided by Dr. Anthony W.S. Chan, Emory). The shRNA expressing lentivectors were prepared by Cellogenetics, Inc. (Gaithersburg, MD) and 25 μg of the scramble shRNA (5'-GTTCTCCGAACGTGTCACG-3') lentivector or the ROCK2 shRNA (5'-CAATGAAGCTTCTTAGTAA-3') expression lentivector was used. The media was replaced 24 hours following the transfection with the 3 plasmids and the supernatant was harvested 48 hours later. The supernatant was filtered using a 0.45 mm pore-sized membrane and concentrated by centrifuging at 10,000× g for 2 hours at 4°C. Lentiviral titres were determined by infecting the HEK 293 cells with varying concentrations of the lentivirus in the presence of Polybrene (8 mg/ml; Sigma Aldrich). The N2aAPPSwe.ΔPS1 cells were infected at an MOI of 10 in the presence of polybrene.

72 hrs post transduction with sh ROCK2 or scramble lentivirus, cells were collected in ice-cold PBS, centrifuged at 55× *g *at 4°C for 15 min, and the media were collected and snap frozen lysates prepared as described above. The lysate membrane was analyzed by western blot using pAb369 (APP C-terminal) to detect APP holoprotein (holoAPP) and α-actin were detected with the respective relevant primary antibodies, followed by HRP-conjugated, species-specific secondary antibodies. Media gels were immunoblotted with 6E10 to detect sAPP and Aβ. Signals were detected using enhanced chemiluminescence (Pierce). Digital images were captured using LAS3000 (FujiFilm), and analyzed using Multi Gauge v3.1 software.

### Statistical analysis

Densitometric analysis of western blot bands (D = [B - AU]/mm^2^) was performed using Multigauge v3.1 software (Fujifilm). Levels of ROCK were normalized to GFP and expressed as percent of control. Total Aβ levels were analyzed by western blot and bands were normalized to percent control (empty vector). Absolute Aβ40 and Aβ42 concentrations were quantitatively determined by sandwich ELISA (Wako). Independent samples t-tests (parametric design) were utilized to determine significant mean differences between groups. Significance for t-tests are reported with a p < 0.05 using two-tailed tests with an α-level of 0.05. All statistical analysis was performed using SPSS v18.0 (SPSS Inc, Chicago, IL).

## Abbreviations

AD: Alzheimer's disease; APP: Amyloid precursor protein; Aβ: Amyloid beta; *TGN*: *trans *Golgi Network; PKC: Protein kinase C; PKA: Protein kinase A; CK2; casein kinase 2; ROCK: rho associated coiled coil kinase; PP1: protein phosphatase 1; PP2A protein phosphatase 2A; sAPPα: soluble amyloid precursor protein alpha; sAPPβ: soluble amyloid precursor protein beta; sSorL1: soluble SorL1; CTF: C terminal fragment; BACE: beta amyloid cleavage enzyme; AICD: amyloid intracellular domain; OA: okadaic acid; SDS PAGE: sodium dodecyl sulfate polyacrylamide gel electrophoresis; CIP: calf intestine alkaline phosphatase; Vps10: vacuolar protein sorting 10; GPCRK1: G protein coupled receptor kinase 1; MEF: mouse embryonic fibroblast; N2a: Neuroblastoma; HEK: human embryonic kidney; MOI: multiplicity of infection; ELISA: enzyme linked immunosorbant assay

## Competing interests

The authors declare that they have no competing interests.

## Authors' contributions

RL and SG conceived the strategic approach and wrote the paper, MEE and SS additionally contributed to the strategic approach and editing of the manuscript. RL and JWG performed the experiments. All authors have read and approved the manuscript. The work was funded by P01AG10491 to SG and P50AG05138 to Mary Sano, and AG025161 and Alzheimer's association to SAS.
